# Diet diversity score might be associated with reproductive health in women and infant outcomes: a systematic review

**DOI:** 10.1017/jns.2024.81

**Published:** 2025-01-02

**Authors:** Paniz Ahmadi, Niloofar Bayat, Behnood Abbasi

**Affiliations:** Department of Nutrition, Electronic Health and Statistics Surveillance Research Center, Science and Research Branch, Islamic Azad University, Tehran, Iran

**Keywords:** Anemia, Birth weight, Diet diversity, Pregnancy outcomes, Reproductive health

## Abstract

Lifestyle and diet may affect the reproductive cycle. A dietary index called Diet Diversity Score (DDS) may be related to various reproductive outcomes. The present review aims to look over and conclude the prior studies on the relationship between the diversity of food ingredients and issues related to reproductive health and pregnancy. In the case of this relationship, our findings can increase clinical knowledge and help recommend a well-balanced diet for the target group. A comprehensive search was performed in major databases such as PubMed, Google Scholar, Web of Science, Scopus, and Scientific Information Database until March 2024. This research was combined with a search of Elsevier and SpringerLink databases, which led to the inclusion of relevant articles in this review. Our study was conducted based on 27 articles from 2012 to 2023, all containing a possible link between dietary diversity and reproductive complications. The Newcastle-Ottawa Scale quality assessment was used to evaluate the quality of included studies. Due to our results, a higher score in DDS, which led to an increased intake of major nutrients and a greater variety of foods, was correlated with a lower risk of reproductive health disorders such as polycystic ovary syndrome, maternal anaemia, and maternal bone status, as well as a reduced likelihood of certain birth outcomes, including low-birth weight infants, Apgar score and congenital heart defect. These findings highlight the importance of improving the DDS for maternal and infant health.

## Introduction

Reproduction is an essential life cycle phase producing new individuals with a unique genome.^(1)^ Currently, many people are influenced by reproductive problems during their lives. According to the World Health Organization report,^(2)^ around 17.5% of the adult population, approximately 1 in 6 worldwide, experience fertility difficulties, demonstrating that this is a major health challenge.^(3)^ Furthermore, according to Njagi *et al.*, the incidence of infertility among couples of reproductive age ranges from 12.6% to 17.5% globally, with almost higher prevalence rates in some regions, such as the Western Pacific, the Americas, Africa, and Europe.^(4)^ Additionally, people’s pockets largely cover fertility treatment costs, and its high financial costs often prevent people from accessing the treatments. For instance, median US costs per IVF cycle or birth were predicted to be about $9,226 and $56,419, respectively, by 2011.^(5)^

Moreover, according to previous studies, reproduction was related to anaemia, infant birth weight, and quality of diet, such as diet diversity, lifestyle, and physical activity.^(6–9)^

Diet diversity is defined as the number of food group items such as cereals, vegetables, fruits, legumes, meat/fish/egg, and dairy products consumed over 24 hours.^(10)^ Diet diversity is considered an essential element of a balanced diet and is also suggested to indicate overall diet quality. Due to most dietary guidelines in the US and globally, increasing the variety of foods across food groups is recommended as it is thought to ensure sufficient intake of key nutrients and promote health.^(11)^ According to the Food Guide Pyramid, there are five main groups to score dietary diversity. Each of the five food sets has a maximum diversity score of two out of the ten possible score points. The total food diversity score is the sum of the scores of the five main groups.^(12)^

Based on the literature, several studies were conducted on the relationship between a diversified diet and reproductive problems despite conflicting results. For instance, Zerfu *et al.* reported that a score of ≥ 4 in dietary diversity food groups during pregnancy was correlated with a lower possibility of maternal anaemia, infants with low-birth weight, and preterm birth, which is explained by the consumption of more nutrient-dense foods such as animal-source foods, fruits, and vegetables, and improved antioxidant and fibre intakes in the adequate DDS group.^(13)^ While Yang *et al.* observed maternal dietary diversity, their associations with inappropriate gestational weight gain and adverse birth outcomes.^(14)^ This inconsistency might be due to differences in study design, participants’ socio-economic status, geographic location, and eating habits.^(15)^

According to our findings, the dietary diversity score and reproduction may probably be related despite the conflicting results. Thus, this systematic review evaluated the articles in this field to determine whether dietary diversity is linked to poor reproductive health and infant outcomes. To the best of our knowledge, this was the first systematic review examining this relationship.

## Methods

This systematic review was carried out based on PRISMA guidelines for Systematic Reviews and Meta-Analyses.^(16)^ This protocol was registered in PROSPERO under the registration number CRD42024548428.

Our research was performed using the following major databases: PubMed, Google Scholar, Web of Science, Scopus, and ProQuest. Moreover, publisher databases, mainly Elsevier, Springer, and Wiley, were also reviewed until March 2024 for possible relevant studies. This search was supplemented by covering the references of all articles for any probably related articles.

All relevant articles were retrieved using a combination of Mesh and non-Mesh terms and keywords like: ‘Pregnancy’, ‘Reproduction’, ‘Infant’, ‘Maternal Dietary Diversity’, ‘Pregnancy Complications’, ‘Reproductive Health’, ‘Diet Diversity Score’, ‘Low Birth Weight’, and ‘Small for Gestational Age’, ‘Polycystic Ovary Syndrome’, ‘Gestational Weight Gain’, ‘Preterm Birth’, ‘Birth Outcomes’, and ‘Apgar Score’.

## Inclusion and exclusion

After screening the titles and abstracts, all related articles addressing the relationship between diet diversity score and reproductive health or different infant outcomes were included to be assessed in the current study. Furthermore, no language restrictions or restrictions regarding the quality of studies were imposed. No limitation criteria were assigned to the study’s publication date or geographic region. In addition, the database search results were classified, duplicate articles were removed using EndNote software, and articles with irrelevant titles and abstracts were excluded. Overall, observational studies were covered, while review articles and experimental and animal studies were ruled out from further assessment.

## Quality assessment

To evaluate the methodological quality and bias risk, each included study was checked out using The Newcastle-Ottawa Scale (NOS) quality instrument. The NOS quality assessment is based on a star rating system for each response, allowing a maximum of seven stars for cross-sectional studies and nine stars for case-control and cohort studies. This assessment consists of three main subscales: Selection, Comparability, and Outcome/Exposure.

**Figure 1.**
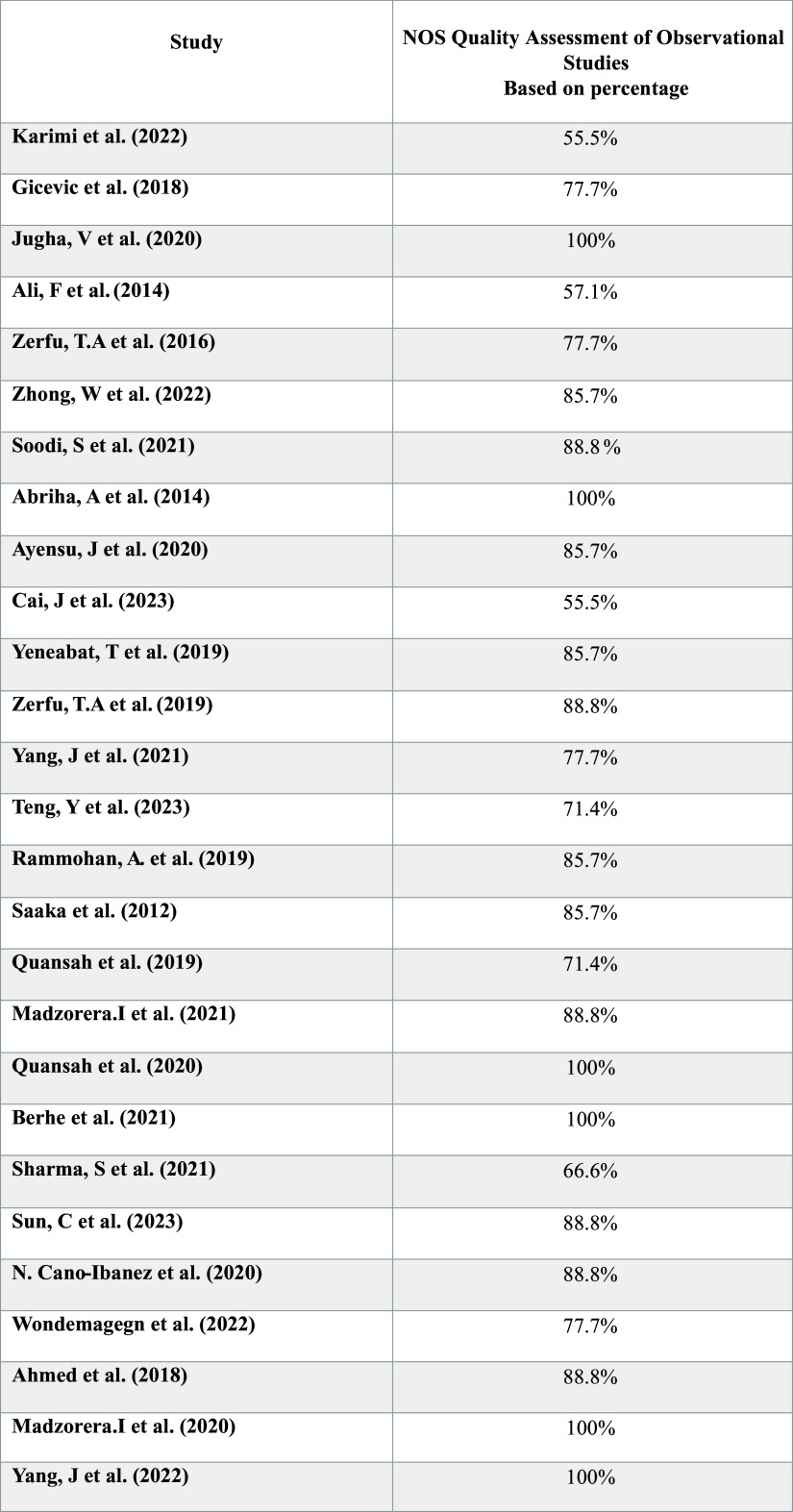
NOS Quality Assessment of 27 observational studies.

Two authors approved the evaluation process. The total star points were then converted into percentages, as illustrated in Fig. 1.^(17)^

## Results

**Figure 2.**
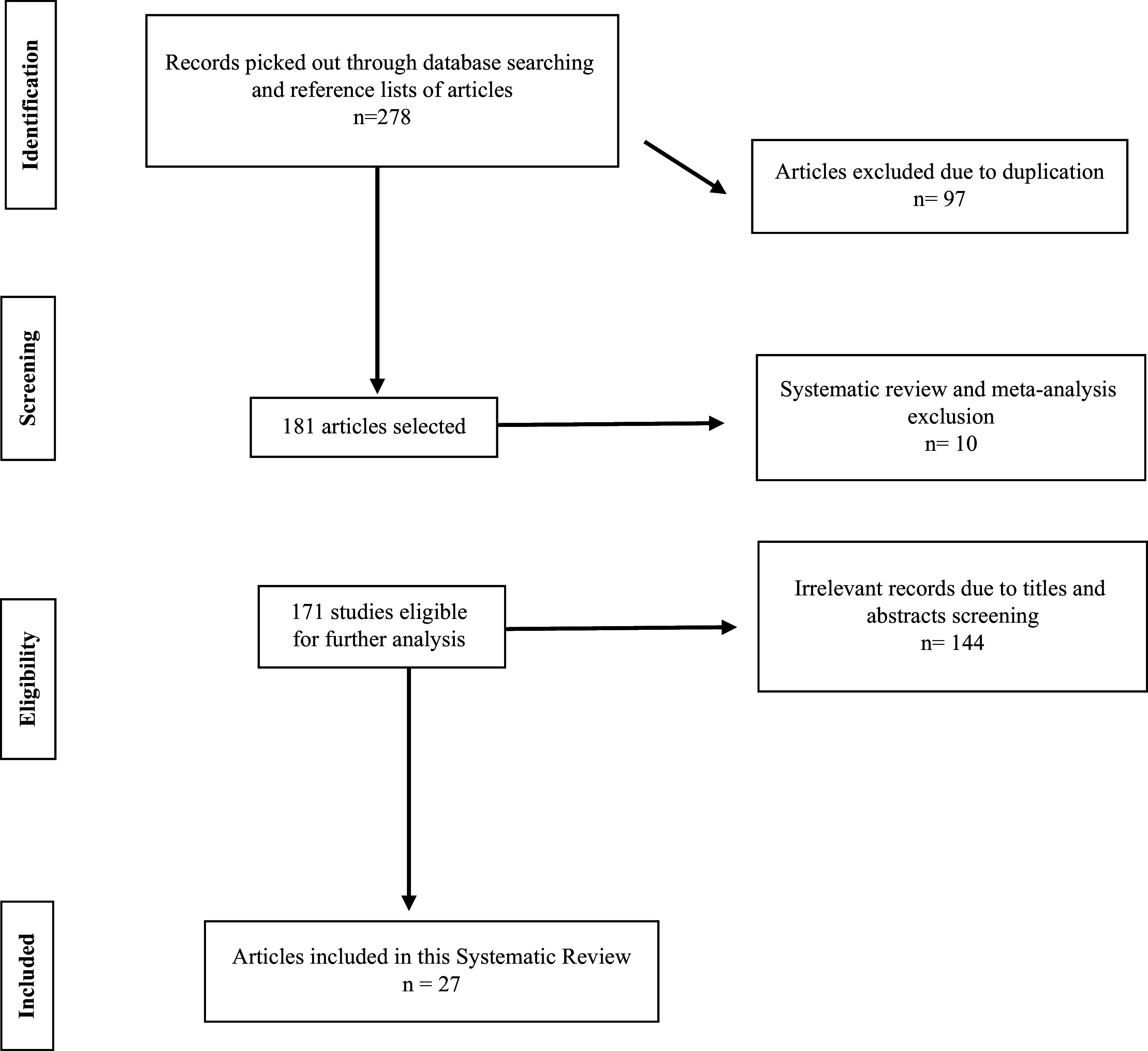
Flow diagram for illustrating the literature search and selection process.

The primary search retrieved 278 articles from the databases mentioned above. According to the inclusion criteria, duplication, and abstract screening, 27 articles between 2012 and 2023 were added to the final analysis (Fig. 2). They all assessed the correlation between diet diversity score and reproductive issues.

The cited articles provided evidence from eleven countries, including the United States of America, Spain, Tanzania, Sri Lanka, Iran, Ethiopia, Ghana, China, Cameroon, Pakistan, and India. Also, the population size of these studies ranged between 200 and 7553 women. Regarding the study design, 11 articles had a cohort design, five had a case-control design, and the remaining 11 were cross-sectional studies.

The articles used various methods for assessing dietary diversity scores, such as FAO MDDW (Minimum Dietary Diversity for Women),^(18)^ Women Dietary Diversity Score (WDDS),^(19)^ and the methodology developed by Kant *et al.*.^(20)^ Additionally, Soodi *et al.* utilised the food guide pyramid approach for DDS measurement.^(21)^ Rammohan *et al.* implemented the DDI method to measure DDS.^(22)^ Furthermore, Saaka *et al.* used the Individual Diet Diversity Score (IDDS) method to measure DDS.^(23)^ Zerfu *et al.* also adopted the Women Individual Dietary Diversity (WIDD) method in their DDS measurements.^(24)^

The articles included were categorised into two main sets: ‘Reproductive Health’ (Table 1) and ‘Infant Outcomes’ (Table 2).

**Table 1.** Summary of twelve selected articles assessing the relationship between diet diversity score and Reproductive Health

Author(s)/ Year	Country	Study Design	Study population and location	DDS Measurement/Food Groups	Max Number	Mean/ Median DDS	Findings
Karimi, T. *et al.* (2022)^(25)^	Iran	Cohort	585 pregnant women in Tehran	Kant *et al.* method^a^/five food groups of the pyramid	10	Before pregnancy: 5.31 ± 1.11, During: 5.23 ± 1.42	A higher dietary diversity and nutritional adequacy before and during pregnancy may cause a higher micronutrient intake, leading to better neonatal outcomes.
Gicevic, S. *et al.* (2018)^(26)^	United States	Cohort	41101 women US nurse without major chronic disease or GDM/HDPs	MDDW method^b^/10 food groups	10	2	The food-based diet diversity score did not predict GDM or HDPs.
Jugha, V. *et al.* (2020)^(27)^	Cameroon	Cross-Sectional	1014 pregnant women in the Mount Cameroon area	MDDW method^b^/10 food groups	10	3.57 ± 0.82	A diverse diet rich in micronutrients had a positive effect on haemoglobin levels and will prevent anaemia.
Ali, F. *et al.* (2014)^(28)^	Pakistan	Cross-Sectional	350 pregnant women at Pakistan Institute of Medical Sciences, Islamabad	MDDW method^b^/10 food groups	10	6.17 ± 0.99	Increasing dietary diversity may lead to weight gain in pregnant women, which is associated with increased food intake and improved nutritional status for both mother and infant.
Zerfu, T. A. *et al.* (2016)^(13)^	Ethiopia	Cohort	374 pregnant women of Arsi zone, Oromia region, Ethiopia	WDDS method^c^/nine food groups	9	Not Reported	Pregnant women in the inadequate DDS group had a higher risk of anaemia, LBW, and PTB due to lower energy and nutrient intake.
Zhong, W. *et al.* (2022)^(29)^	China	Cross-Sectional	775 pregnant women aged 18 years old or older in urban China	MDDW method^b^/10 food groups	10	6.61 ± 1.53	A diversified diet, including
							animal foods and dairy products rich in calcium, vitamin D, and protein were correlated with better bone status.
Soodi, S. *et al.* (2021)^(30)^	Iran	Case-control	494 participants (203 cases and 291 controls)	Food Guide Pyramid^e^/ five food groups of the pyramid	10	5.19 ± 1.19 for the cases and 5.51 ± 1.19 for the controls	PCOS women had lower consumption of meat and vegetables and higher intake of high-glycemic index foods and sugar-rich foods. An enhanced DDS will reduce the risk of PCOS.
Abriha, A. *et al.* (2014)^(31)^	Ethiopia	Cross-Sectional	619 pregnant women in Mekele, Northern Ethiopia	Single 24-hour recall/Not reported	High DDS ≥ 6	4.9	Food frequency, dietary diversity, and red meat consumption due to increased haemoglobin concentrations and heme iron levels significantly affect anaemia in pregnant women.
Ayensu, J. *et al.* (2020)^(32)^	Ghana	Cross-Sectional	379 pregnant women in the Ashanti region	WDDS method^c^/nine food groups	10	3.81 ± 0.7	Dietary diversity positively impacted haemoglobin levels, associated with a lower odd of anaemia.
Cai, Z. *et al.* (2023)^(33)^	China	Cohort	969 pregnant women from rural townships in Western China	WDDS method^c^/nine food groups	9	6.2 ± 1.39	Improving dietary diversity leads to higher consumption of nutrient-dense foods incredibly bioavailable heme iron, vitamin A, and vitamin C sources, reducing maternal anaemia.
Yeneabat, T. *et al.* (2019)^(34)^	Ethiopia	Cross-Sectional	834 pregnant women in East Gojjam Zone, Northwest Ethiopia	MDDW method^b^/10 food groups	10	3.68 ± 2.10	The consumption of less diversified food, decreased meal frequency, and food groups consumed by non-educated pregnant women were common.
Zerfu, TA. *et al.* (2019)^(24)^	Ethiopia	Cohort	432 pregnant women in Arsi Zone, Central Ethiopia	WIDD method^**d**^/nine food groups	9	Not Reported	Low DDS and not consuming animal-source foods, along with pre-existing anaemia, were associated with a risk of anaemia at term.

a **Kant** ***et al.*** ^(20)^ method: this method includes five food groups such as 1) dairy, 2) meat, 3) grain, 4) fruit, 5) vegetables.

b **MDDW** ^(18)^method: Minimum Dietary Diversity for Women Food groups: It includes the 10 following food groups: 1) starchy staples, 2) pulses, 3) nuts and seeds, 4) dairy, 5) meat, poultry, and fish, 6) eggs, 7) dark-green leafy vegetables, 8) other vitamin-A rich fruits and vegetables, 9) other vegetables, and 10) other fruits.

c **WDDS** ^(19)^ method: Women Dietary Diversity Score Food groups: The foods were categorised into nine food groups: 1) cereals, roots, and tubers; 2) vitamin A– rich fruit and vegetables; 3) other fruit; 4) other vegetables; 5) legumes and nuts; 6) meat, poultry, and fish; 7) fats and oils; 8) dairy; and 9) eggs.

d **WIDD** ^(24)^ method: Women Individual Dietary Diversity Food groups following nine food groups to calculate the WIDD score: 1) cereals, roots, and tubers; 2) dark-green leafy vegetables; 3) vitamin A-rich fruits and vegetables; 4) other fruit and vegetables; 5) legumes and nuts; 6) meat, poultry, and fish; 7) organ meat; 8) dairy; and 9) eggs.

e **Food Guide Pyramid** ^(21)^: 5 main groups, including 1) grains/bread, 2) vegetables, 3) fruits, 4) meats, and 5) dairy products, were used.

**Table 2.** Summary of fifteen/sixteen selected articles assessing the relationship of dietary diversity with Infant Outcomes

Author (s)/Year	Country	Study Design	Study population and location	Measurement of DDs/Food Groups	Max Number	Mean/Median DDS	Findings
(Karimi, T. *et al.*) (2022)^(25)^	Iran	Cohort	585 pregnant women in Tehran	Kant *et al.* method^a^/five food groups of the pyramid	10	Before pregnancy: 5.31 ± 1.11, During: 5.23 ± 1.42	A higher dietary diversity and nutritional adequacy before and during pregnancy may cause higher micronutrient intake, leading to better neonatal outcomes.
Yang. J, *et al.* (2021)^(35)^	China	Case-Control	474 cases and 948 control in Xi’an City, Northwest China	MDDW method^b^/10 food groups	10	5 for the cases and 6 for the controls	Adequate DDS during pregnancy might be associated with a lower risk of CHD in offspring through improved antioxidant status.
Madzorera.I., *et al.* (2020)^(36)^	Tanzania	Cohort	7553 HIV-negative pregnant women from Tanzania	MDDW method^b^/10 food groups	10	3	A diversified maternal diet rich in energy and protein intake leads to preventing SGA and LBW infants.
Teng. Y, *et al.* (2023)^(37)^	China	Cross-Sectional	6805 Chinese pregnant women	MDDW method^b^/10 food groups	10	6.11 ± 1.97	Maternal dietary diversity full of animal-based foods and high-quality proteins was positively associated with neonate birth weight.
Yang, J. *et al.* (2022)^(14)^	Tanzania	Cohort	1190 pregnant women in urban Tanzania	MDDW method^b^/10 food groups	10	4.2 ± 1.9	A poor maternal diet diversity would fail to meet the nutrition required for mother and foetus, thus leading to a higher risk of different birth outcomes.
Rammohan, A, et.al (2019)^(22)^	India	Cross-Sectional	230 women in Lucknow	DDI method^c^/eight food group	4.063	0 ± 1	Lack of access to food and low diversity in food intake leads to maternal malnutrition and LBW infants.
Saaka (2012)^(23)^	Ghana	Cross-Sectional	524 pregnant women in Ghana	FAO IDDS method^**d**^/11 food groups	11	9.1 ± 1.4	Higher maternal dietary diversity results in more significant nutrient transfer across the placenta, leading to a lower risk of LBW infants.
Quansah. *et al.* 2019)^(38)^	Ghana	Cross-Sectional	420 mothers	MDDW method^b^/10 food groups	10	Not Reported	A diverse diet during pregnancy provides essential nutrients for foetus growth, leading to a higher Apgar score.
Madzorera. I, *et al.* 2021)^(39)^	Uganda	Cohort	3291 women and infant pairs from Uganda’s population	MDDW method^b^/10 food groups	10	During pregnancy: 3.4 At all other times: 3.1	Maternal diet diversity and nutrient deficiencies during pregnancy may predispose the foetus to experience nutrient deficits that can affect its growth.
Quansah. *et al.* 2020)^(40)^	Ghana	Cross-Sectional	420 mothers	MDDW method^b^/10 food groups	10	Not Reported	Higher dietary diversity provides adequate micronutrients that are important for foetal development and may be protective of LBW.
Berhe *et al.* 2021)^(41)^	Ethiopia	Cohort	500 pregnant women in Tigray regional state	MDDW method^b^/10 food groups	10	Not Reported	Poor diet diversity may cause multiple micronutrient deficiencies, negatively impacting birth weight.
Sharma S *et al.* 2021)^(42)^	India	Case-control	157 mothers with and 214 without an LBW child in India.	MDDW method^b^/10 food groups	10	Mothers with LBW child: 2.2 ± 1.3 Mothers without LBW child: 2.7 ± 1.7	A less diverse diet may limit nutrient availability to transfer to the foetus and affect birth outcomes.
Sun, C *et al.* (2023)^(43)^	China	Cohort	560 mothers in Southwestern China	MDDW method^b^/10 food groups	10	Not Reported	Inadequate diet diversity was associated with SGA infants through disruptions in the functioning of the placenta.
N. Cano-Ibanez *et al.* (2020)^(44)^	Spain	Case-control	518 cases and controls of pregnant women in Eastern Andalusia	Kant *et al.* method^a^/five food groups of the pyramid	10	Not Reported	High total DDS and dairy products were associated with a lower risk of SGA newborn due to the inclusion of calcium and high biological value proteins.
Wondemagegn *et al.* (2022)^(45)^	Ethiopia	Cohort	416 pregnant mothers in East Gojjam, Ethiopia	MDDW method^b^/10 food groups	10	4.08 ± 1.64	An elevated score in diet diversity during pregnancy may serve essential nutrients necessary for a newborn’s growth and lead to improved birth outcomes.
Ahmed *et al.* 2018))^(46)^	Ethiopia	Case-control	95 cases and 191 controls in Dessie Town, Northeast Ethiopia	MDDW method^b^/10 food groups	10	Not Reported	Inadequate maternal dietary diversity due to micronutrient deficiencies was a determinant of LBW infants.

a **Kant** ***et al.*** ^(20)^ method: this method includes five food groups such as 1) dairy, 2) meat, 3) grain, 4) fruit, and 5) vegetables.

b **MDDW** ^(18)^ method: Minimum Dietary Diversity for Women Food groups: It includes the ten following food groups: 1) starchy staples, 2) pulses, 3) nuts and seeds, 4) dairy, 5) meat, poultry, and fish, 6) eggs, 7) dark-green leafy vegetables, 8) other vitamin-A rich fruits and vegetables, 9) other vegetables, and 10) other fruits.

c **DDI** ^(22)^method: Dietary Diversity Index: including eight main groups: 1) grains, 2) pulses, 3) flour types, 4) sweet, 5) egg, 6) milk products, 7) fruits, and 8) vegetables.

d **IDDS** ^(23)^ method: Individual Dietary Diversity Score: including 11 food groups: flesh meats (i.e. beef, pork, lamb, goat, poultry), fish, eggs, milk and milk products, organ meat (e.g. liver, kidney), legumes, cereals, roots & tubers, dark-green leafy vegetables, vitamin A rich fruits and fats & oils.

### Reproductive health

The following group assessed in this study is women’s reproductive health, which includes conditions such as anaemia, nutritional and bone status, polycystic ovary syndrome, and metabolic disorders.

#### Maternal anaemia

Six out of 27 studies analysed the effect of a diversified diet on the prevalence of anaemia, all of which indicated a beneficial impact of higher DDS on maternal anaemia and, thus, a beneficial impact on managing this condition.^(13,24,27,31–33)^

#### Nutritional status

In sum, four out of 27 articles examined the ratio of nutrient adequacy in women’s diets. Four papers found that a higher score in dietary diversity was correlated with greater nutritional status for women of reproductive age.^(25,28,29,34)^

#### Bone status

One out of 27 articles examined the effect of a varied diet on women’s bone status. Zhong W. *et al.* reported that a diversified diet was positively associated with better bone status, recommending the importance of modifying diet diversity for pregnant women.^(29)^

#### Polycystic ovary syndrome

Of the 27 studies, Soodi *et al.* found a link between diet diversity score and the incidence of polycystic ovary syndrome in women of reproductive age, showing a significant association with higher scores and lower odds of PCOS.^(30)^

#### Metabolic disorders

Out of 27 studies, Gicevic S. *et al.* identified the association between diet diversity score and different metabolic disorders. Gicevic S. *et al.* stated that the food-based dietary diversity score did not predict gestational diabetes mellitus (GDM) and hypertensive disorders of pregnancy (HDPs).^(26)^

### Infant outcomes

Infant outcomes are the other set, containing articles on subjects such as low-birth weight and preterm birth infants (LBW), Apgar score, small for gestational age foetuses (SGA), and congenital heart defect.

#### Low birth weight and preterm birth infants

Eleven out of 27 articles expressed the correlation of diet diversity score with low-birth weight and preterm birth infants. The findings indicated that a greater maternal diet diversity was associated with lower rates of low-birth-weight infants, a significant relationship consistently observed across all eleven studies.^(14,22,23,36,37,39–42,45,46)^

#### Small for gestational age (SGA)

Two studies evaluated the relationship between small for gestational age infants and diet diversity scores from the 27 articles. These studies found that women with a higher diet diversity score presented a lower SGA risk.^(43,44)^

#### Apgar score

Among the 27 papers, Quansah D. Y. *et al.* assessed the correlation between diet diversity and infant Apgar scores. Indeed, they reported a positive impact of increasing diet diversity on the Apgar score. Most mothers who gave birth to babies with a standard Apgar score had a more diversified diet.^(38)^

#### Congenital heart defect

Of the total of 27 studies, Yang *et al.* was the only study that examined the effect of diet diversity on the neonatal heart. Thus, it was concluded that an adequate diet diversity score during pregnancy might be related to a reduced chance of congenital heart defects.^(35)^

## Discussion

To the best of our knowledge, this is the first systematic review to investigate the correlation between diet diversity score and reproductive health among women, as well as different infant outcomes, aimed at updating and concluding the prior studies.

The diet diversity score was related to maternal nutritional status. Karimi *et al.* pointed out that women with a higher score in diet diversity had an increased mean adequacy of different food groups and higher intake in all main components of the DDS, such as cereals and bread, meat, dairy products, vegetables, and fruit groups. As a result, they received more diverse foods. These circumstances may be correlated with different birth outcomes, affecting newborns’ bioavailability of nutrient resources.^(25)^

## Overview of evidence related to women’s reproductive health

In the present study, 12 articles emphasised the significant impact of dietary diversity on women’s reproductive health, particularly highlighting the adverse effects of a low-diversity diet.

According to our assessment, a more diversified diet may be related to a lower incidence of maternal anaemia, which aligns with the findings from the six articles on this topic. Specifically, women who achieve a higher diet diversity score tend to consume more micronutrient-rich foods, such as sources of animal foods, vegetables, and fruits.^(47)^ Conversely, inadequate dietary diversity leads to mineral and vitamin deficiencies, which may affect iron levels and bioavailability.^(48)^ Moreover, meat consumption due to increased haemoglobin concentration and as an essential source of heme iron was another factor that showed a significant relationship with anaemia in pregnant women.^(49)^ Organ meats are an excellent source of iron and vitamin A, while milk and its products also provide substantial amounts of vitamin A. These nutrients are crucial in synthesising red blood cells and haemoglobin.^(50)^ Moreover, meat contains protein with a high biological value, and fruits abundant in ascorbic acid can enhance iron absorption. Likewise, eating less or not eating meat and fruits also leads to inadequate intake of iron, which ultimately causes anaemia.

Furthermore, vegetables provide a valuable source of folic acid, and a lack of folic acid is related to anaemia during pregnancy.^(47)^ Overall, a diverse diet consisting of different food groups rich in micronutrients, particularly iron, vitamin A, vitamin B12, and folic acid, promotes the production of red blood cells and thus prevents anaemia in women of reproductive age.^(27)^ However, dietary practices and cultural taboos may also restrict mothers from consuming available iron-rich foods.^(51)^

In this review, we investigated the associations between diet diversity score and bone status in women. Our results were aligned with Zhong *et al.*,^(29)^ which confirmed that women with a higher diet diversity score had a higher intake of animal-source foods, such as dairy products, eggs, and flesh foods. These are rich sources of nutrients such as calcium, vitamin D, and protein, which are beneficial for peak bone mass, reduced bone resorption, and enhanced bone mineral density.^(52)^ In addition, as stated by Weaver *et al.*, women with a diversified diet had a higher consumption of fruits, vegetables, pulses, and nuts or seeds, which were rich in unsaturated fatty acids and bioactive complexes such as flavonoids that protect in case of oxidative stress and inflammation.^(53)^

In agreement with our assessments, Soodi *et al.* also reported that an elevated diet diversity score was significantly related to a lower incidence of polycystic ovary syndrome. Women with PCOS had more consumption of high-glycemic index foods and a lower intake of vegetables and legumes. As Azadbakht *et al.* and Shishehgar *et al.* claimed, vegetable consumption might be associated with a lower risk of expanding abdominal adiposity and obesity, both of which are the major symptoms of PCOS. It should also be noted that consuming vegetables can improve ovulation by increasing insulin sensitivity.^(54,55)^ Furthermore, Hajivandi *et al.* found that adolescent girls with polycystic ovary syndrome had unhealthy eating habits with a low consumption of meat, fish, and seafood and a high intake of fats full of sugar, unhealthy and fatty foods, which, as a result, cause some problems in women with polycystic ovary syndrome and reproductive issues.^(56)^

Following the results of our study, Liao *et al.* showed that the risk of GDM was inversely associated with fruit intake. This relationship might be explainable by polyphenols and antioxidant complexes found in fruits, such as carotenoids and vitamins E and C. These compounds reduce oxidative stress in cells, interfering with glucose absorption and preventing the development of abnormal glucose tolerance. Moreover, the presence of fibre in fruits and vegetables can delay the absorption of food carbohydrates and avoid a rapid increase in blood sugar.^(57)^ Gicevic *et al.* found that the food-based dietary diversity score did not correlate with GDM or HDPs. The following mechanism can describe this: the DDSs do not consider the specific types of carbohydrates, fats, and forms of animal protein sources in the total diet. Therefore, the inclusion of refined grains, saturated and trans fatty acids, and red and processed meats associated with a higher risk of several different chronic diseases, such as GDM or HDPs, may partly explain the findings from this study.^(26)^

## Overview of evidence related to infant complications

An inappropriate maternal diet with insignificant diversity and quality would not meet the nutritional requirements of the mother and foetus, which could lead to an increased risk of in-pregnancy complications and harmful consequences of childbirth.^(35)^

In line with our assessments, articles also claimed that maternal nutritional status and micronutrient deficiencies during pregnancy may have severe consequences for the health of the developing foetus, thereby affecting birth size and the availability of nutrients to transfer to the foetus.^(58,59)^

In close agreement with Quansah *et al.*, our findings indicated that a diversified diet may be correlated with a reduced risk of infants with low-birth weight.^(40)^

In addition, a low diet diversity score during pregnancy can lead to a lack of essential micronutrients important for foetal growth and development. However, improved micronutrient intake, resulting in greater dietary diversity, can ameliorate infant complications through improved antioxidant and fibre intake.^(60)^ According to Yang *et al.*, the contrasts in maternal diet diversity among mothers with or without low-birth-weight infants, lack of animal protein intake, and inadequate dietary patterns could be the main nutritional complication for Chinese pregnant women.^(61)^

Moreover, lower DDS may develop anaemia, impairing oxygen delivery to the foetus and hence interfering with normal intrauterine development, possibly leading to low-birth weight.^(62)^

Consistent with our assessment with Zerfu *et al.*, the findings of this review showed that diet diversity, an indicator of diet quality, was related to preterm birth and birth weight.^(13)^ According to a prospective cohort study involving 66,000 pregnant women in Norway, ‘prudent’ dietary patterns that included fruits, vegetables, whole-grain cereals, fibre-rich bread, oils, and water reported a lower chance of premature delivery.^(63)^

This current study found that diet diversity score during pregnancy may be positively related to the Apgar score. As Quansah noted, consuming a diverse diet during pregnancy provides essential nutrients for foetal growth and development. Consuming fewer food groups could result in a lack of important nutrients needed during pregnancy for the foetus’s growth and development.^(64)^ Since the Apgar score is related to birth weight, a low-birth-weight newborn probably has higher odds of having a low Apgar score due to poor nutrition throughout gestation.

One of the outcomes investigated in this review was the relationship between DDS and SGA infants. With the same result, Sun *et al.* indicated that having an underweight pre-pregnancy BMI and also an insufficient dietary diversity score increased the mother’s chance of delivering a small for gestational age infant.^(43)^

Besides, as claimed by Cano-Ibanez *et al.*, diet diversity improves nutrient status and provides benefits not offered by supplementation during pregnancy. This analysis suggests that women with a high total DDS and more intake of dairy items had a protective impact against being small for gestational age..^(44)^ Additionally, the findings of Zerfu *et al.* showed a decreased risk of preterm birth and low-birth weight in pregnant women with a high diversity of dairy products,^(65)^ which are a good source of key nutrients, like calcium and high biological value proteins.^(66)^ These nutrients are related to preventing small for gestational age due to their contribution to foetal growth.

As a result of our review, which is supported by Yang *et al.*,^(35)^ a probable mechanism by which an adequate diet diversity score during pregnancy can decrease congenital heart defect risk would be through improving antioxidant status. Likewise, enhanced dietary diversity has been reported to be correlated with reduced oxidative stress in women. Maternal oxidative stress in pregnancy may affect the normal growth of the foetal cardiovascular system.^(35,67,68)^ These findings highlight the importance of an increase in diet diversity score, which has a practical impact on women’s reproductive health and infant healthiness.

The present study was conducted to conclude previous studies on the relationship between the diversity of food ingredients and reproductive issues, which reported a positive relationship between higher DDS and reduced risk of reproductive health and infant issues. These findings can increase clinical and pre-clinical knowledge to recommend a diversified diet for the target group.

Considering that the present systematic review is a qualitative study, we conducted a review incorporating different study designs, including all observational studies, to draw conclusions and obtain reliable results from the relevant studies. Furthermore, no restrictions were placed on the publication date or language in our study.

Lastly, the articles included used various methodologies reporting DDS; therefore, quantitative analysis regarding different score calculation methods, was not possible.

## Conclusion

Due to our findings, a higher dietary diversity score was correlated with a lower probability of reproductive disorders, including maternal anaemia and polycystic ovary syndrome, and a better status in nutrition and bone health. Moreover, a diversified diet may be correlated with a reduced risk of adverse birth outcomes, such as preterm delivery, low-birth weight infants, congenital heart defect, and a higher Apgar score. Likewise, results showed that DDS may not be correlated with GDM and HDPs. However, further studies are required in this area.

## Abbreviations

**DDS:** Diet Diversity Score; **GDM:** Gestational Diabetes Mellitus; **HDPs:** Hypertensive Disorders of Pregnancy; **LBW:** Low Birth Weight; **PCOS:** Polycystic Ovary Syndrome; **SGA:** Small for Gestational Age
